# Supplementary material for: CO2 electrolysis to multi-carbon products in strong acid at ampere-current levels on La-Cu spheres with channels
Source: Nat Commun. 2024 Jun 6;15:4821. doi: 10.1038/s41467-024-49308-8 (PMC11156665; doi:10.1038/s41467-024-49308-8)
Supplement: Supplementary file 1 — Supplementary Information [file 41467_2024_49308_MOESM1_ESM.pdf]

Supporting Information

CO_2 electrolysis to multi-carbon products in strong acid at ampere-current levels on La-Cu spheres with channels

Jiaqi Feng[1,2], Limin Wu[1,3], Xinning Song[1,3], Libing Zhang[1,3], Shunhan Jia[1,3], Xiaodong Ma[1,3], Xingxing Tan[1,3], Xinchen Kang[1,3], Qinggong Zhu[1,3], Xiaofu Sun[1,3]*, Buxing Han[1,3,4]*

1. Beijing National Laboratory for Molecular Sciences, Key Laboratory of Colloid and Interface and Thermodynamics, Center for Carbon Neutral Chemistry, Institute of Chemistry, Chinese Academy of Sciences, Beijing 100190, China

2. College of Chemical Engineering and Environment, China University of Petroleum (Beijing), Beijing 102249, China

3. School of Chemical Sciences, University of Chinese Academy of Sciences, Beijing 100049, China

4. Shanghai Key Laboratory of Green Chemistry and Chemical Processes, School of Chemistry and Molecular Engineering, East China Normal University, Shanghai 200062, China

* To whom correspondence should be addressed. Email: sunxiaofu@iccas.ac.cn and hanbx@iccas.ac.cn

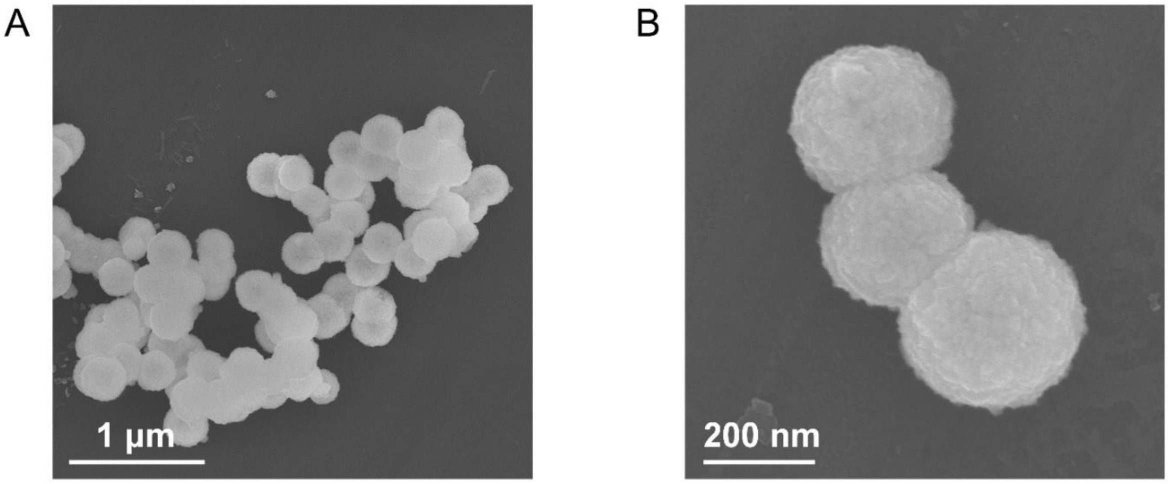

Supplementary Fig. S1 | SEM images of La-Cu_2O HS in (A) low and (B) high magnification.

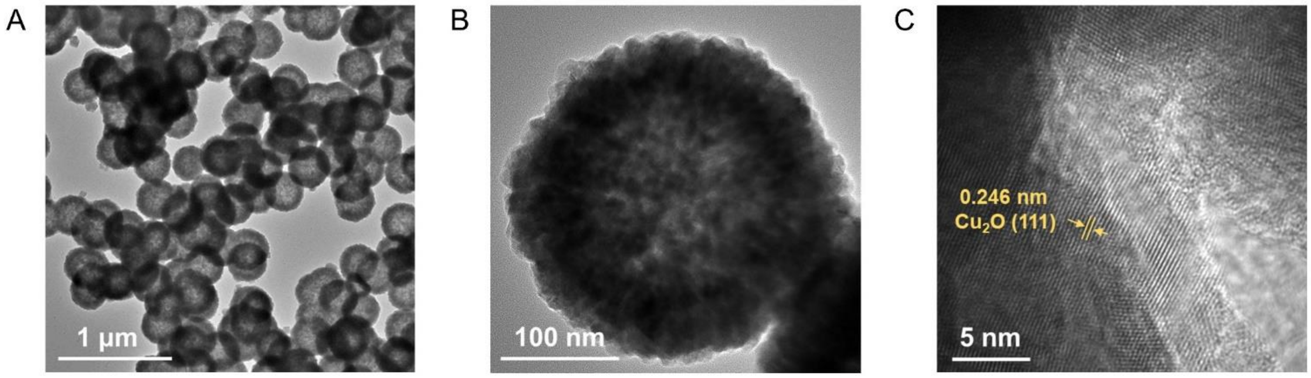

Supplementary Fig. S2 | (A, B) TEM images and (C) HRTEM image of La-Cu_2O HS.

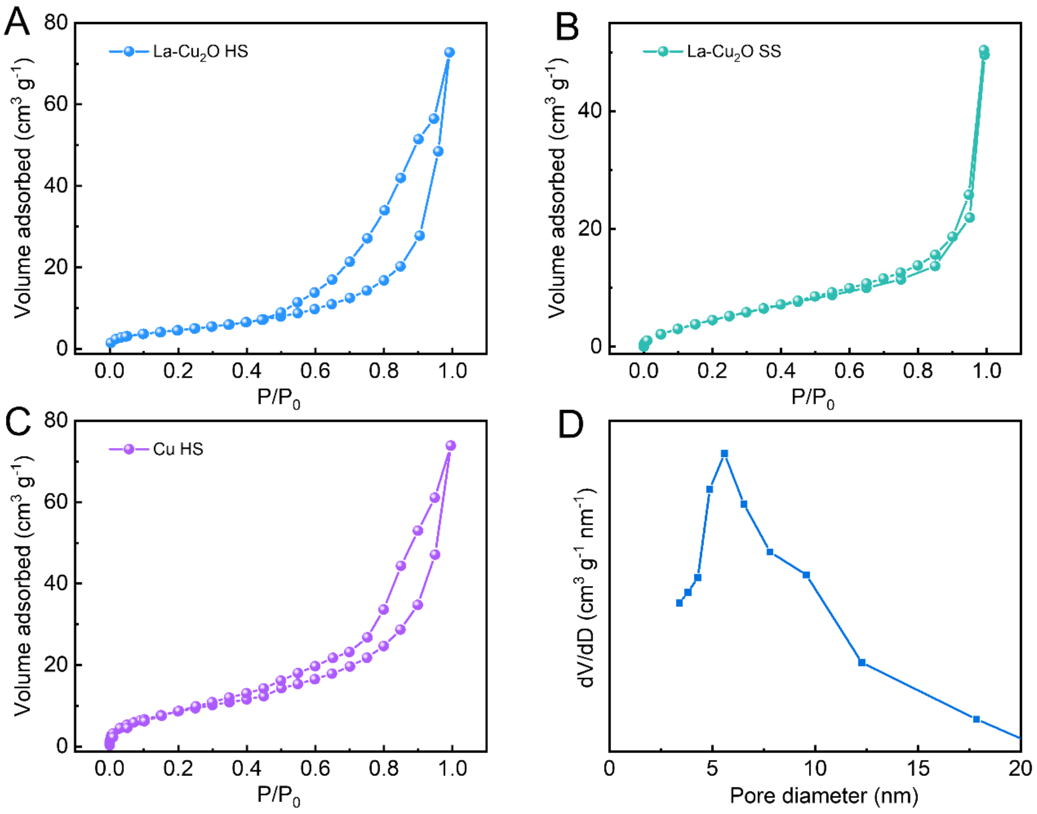

Supplementary Fig. S3 | Nitrogen adsorption-desorption isotherm of (A) La-Cu_2O HS, (B) La-Cu_2O SS, and (C) CuO_x HS. (D) The corresponding BJH pore size distribution of La-Cu_2O HS.

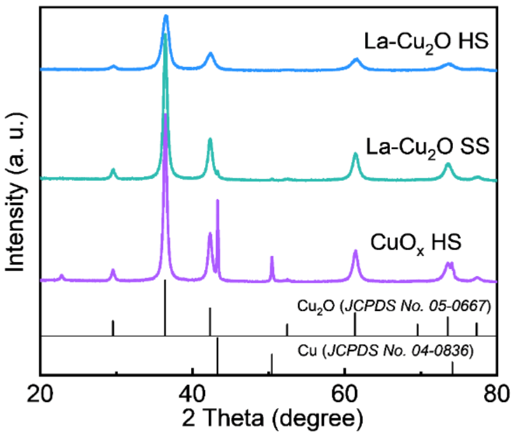

Supplementary Fig. S4 | XRD patterns of La-Cu_2O HS, La-Cu_2O SS, and CuO_x HS.

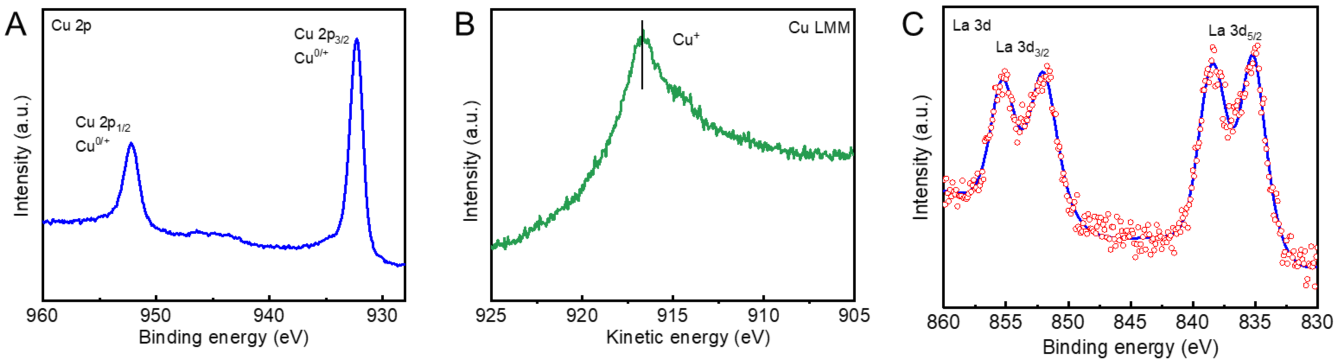

Supplementary Fig. S5 | (A) Cu 2p XPS profile, (B) Cu Auger LMM spectra, and (C) La 3d XPS profile of La-Cu_2O HS.

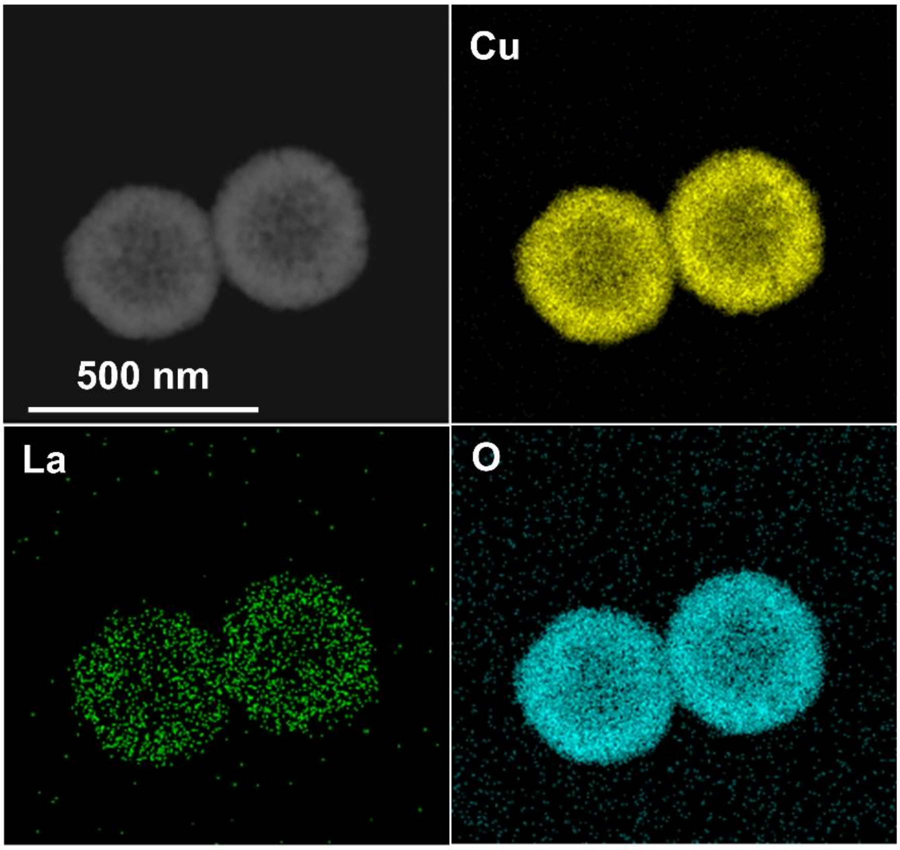

Supplementary Fig. S6 | EDS mapping of La-Cu_2O HS. Yellow, green, and blue represent Cu, La, and O elements, respectively. The results indicate that Cu, La, and O elements are uniformly distributed on La-Cu_2O HS.

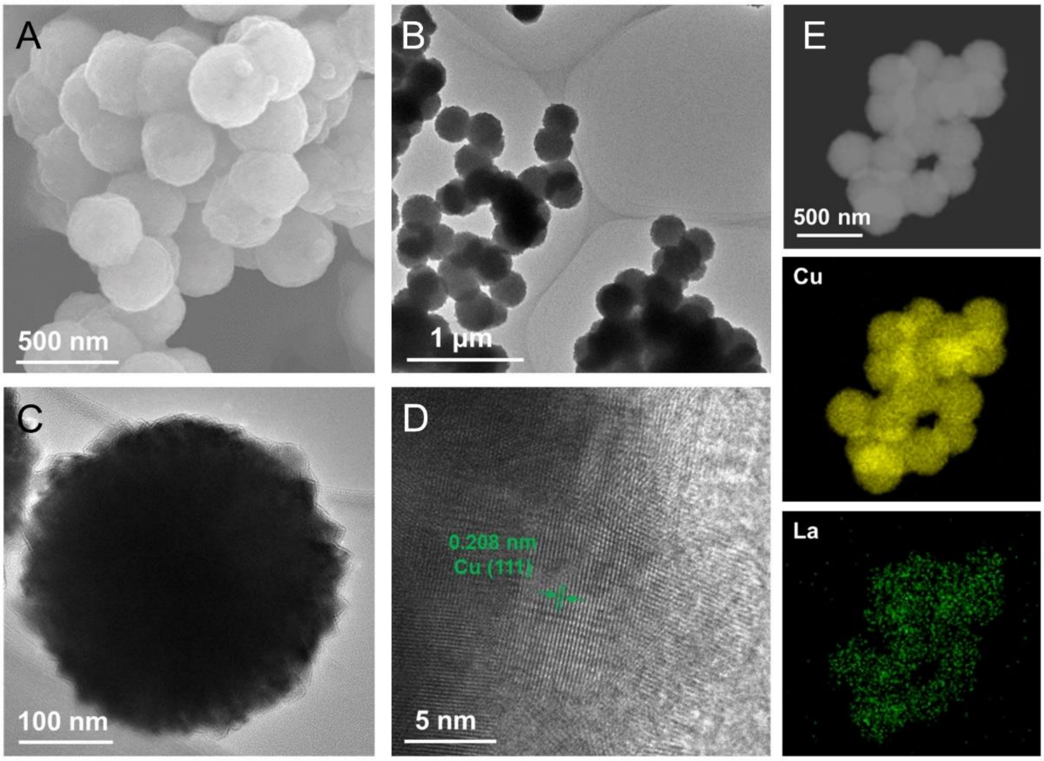

Supplementary Fig. S7 | (A) SEM, (B, C) TEM, (D) HRTEM images, and (E) EDS mapping of La-Cu SS (yellow and green represent Cu and La elements, respectively.).

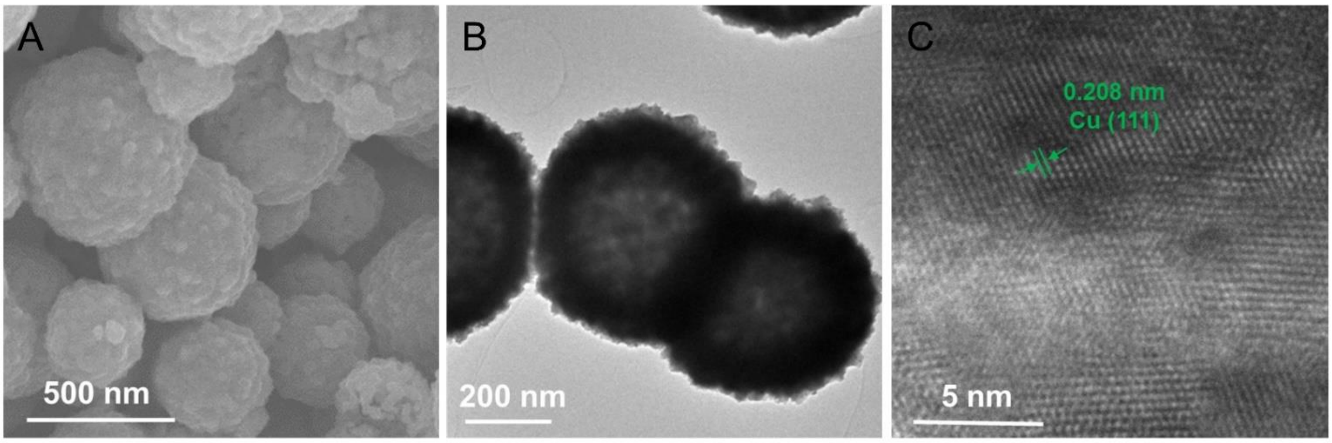

Supplementary Fig. S8 | (A) SEM, (B) TEM, and (C) HRTEM images of Cu HS.

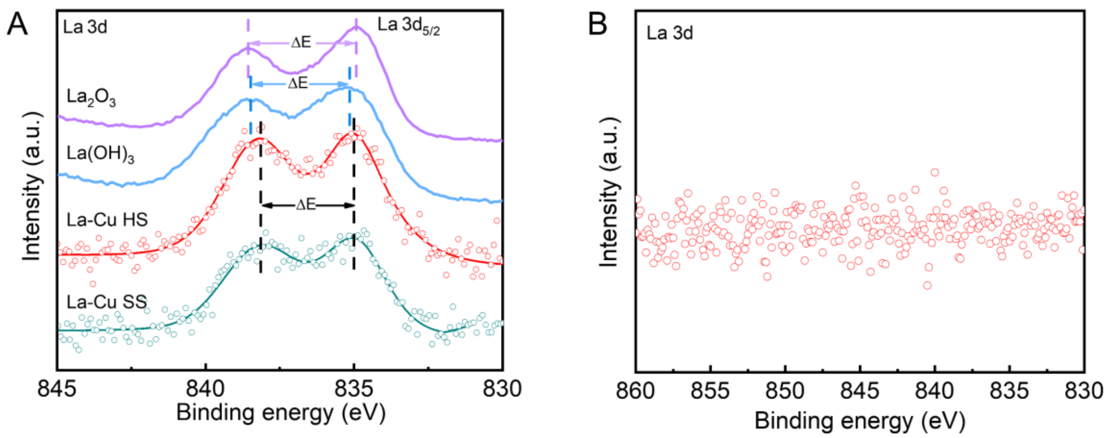

Supplementary Fig. S9 | La 3d XPS profile of (A) La-Cu HS, La-Cu SS, La_2O_3, $La(OH)_3$ and (B) Cu HS.

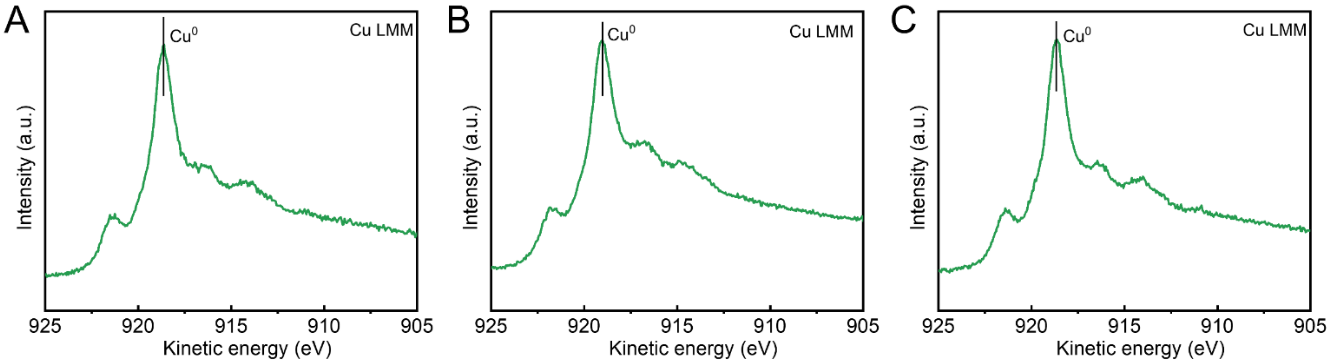

Supplementary Fig. S10 | Cu Auger LMM spectra of (A) La-Cu HS, (B) La-Cu SS, and (C) Cu HS.

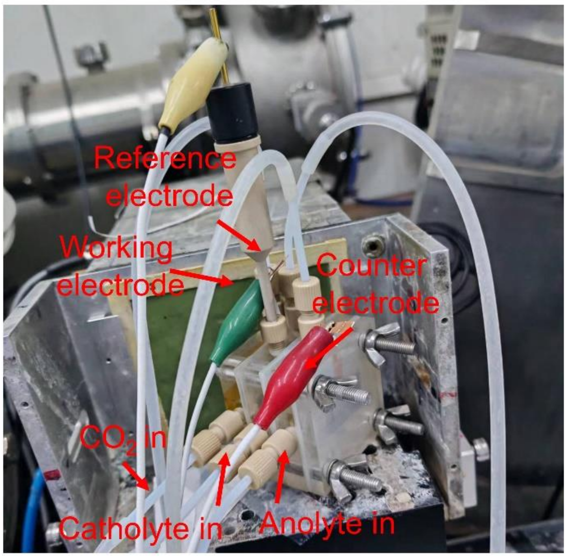

Supplementary Fig. S11 | The optical photo of flow cell used for *in situ* XAS experiment.

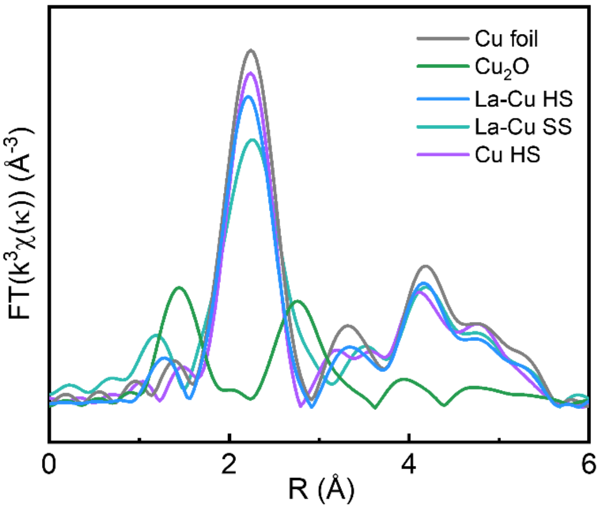

Supplementary Fig. S12 | Fourier transform extended X-ray absorption fine structure spectra of La-Cu HS, La-Cu SS, and Cu HS.

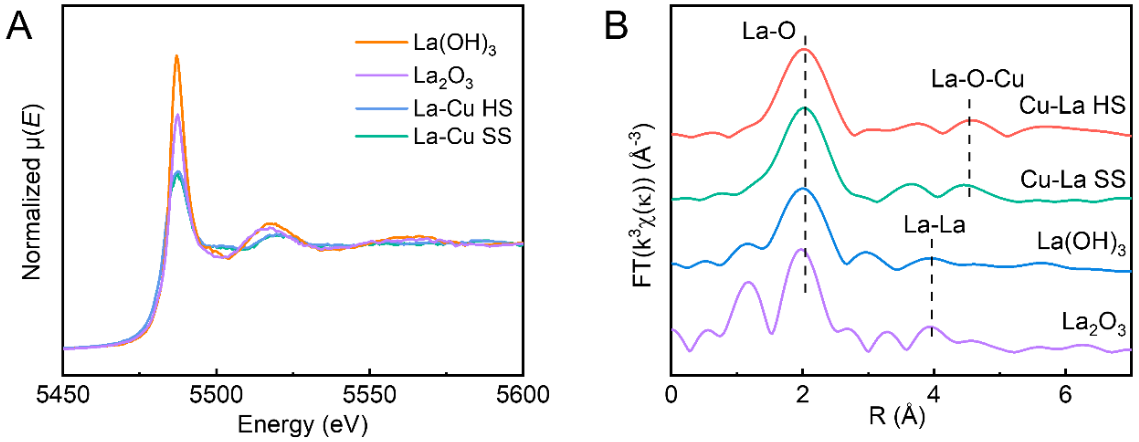

Supplementary Fig. S13 | (A) La L_3-edge XANES spectra and (B) FT EXAFS spectra of La-Cu HS, La-Cu SS, $La(OH)_3$ and La_2O_3.

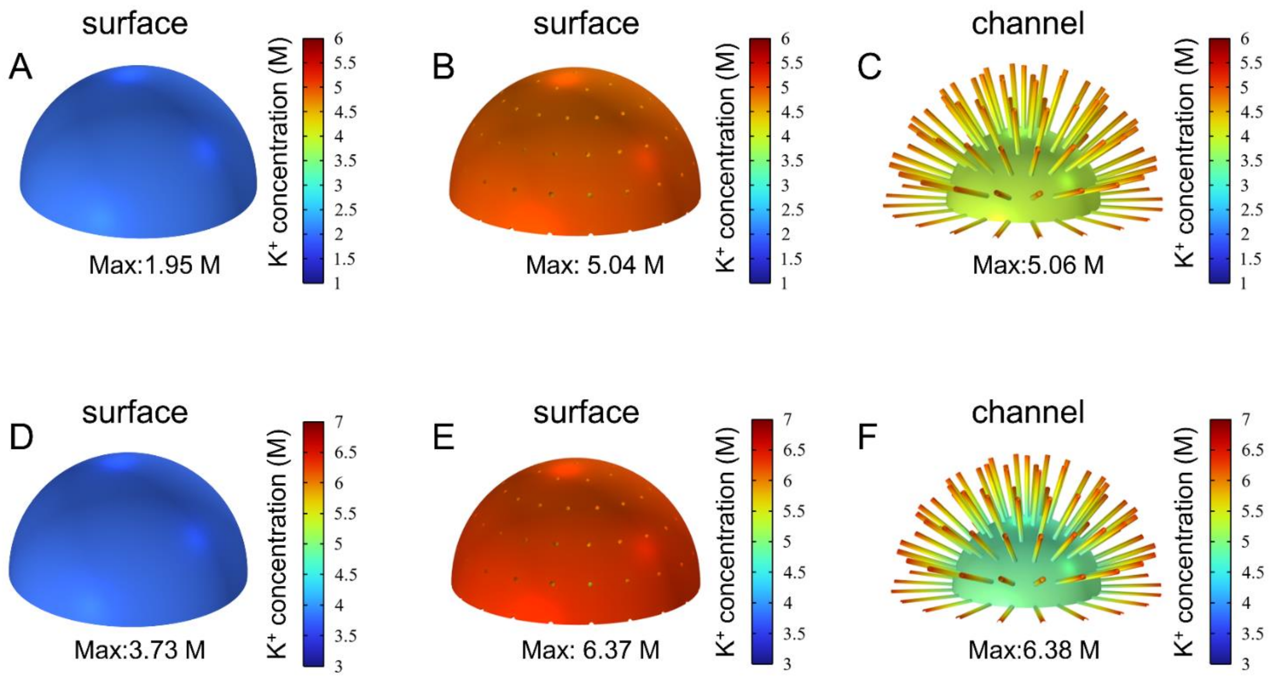

Supplementary Fig. S14 | The K^+ concentration distribution over surface of (A) solid sphere, (B) hollow sphere with channels, and within channels of (C) hollow sphere at -300 mA. The K^+ concentration distribution over surface of (D) solid sphere, (E) hollow sphere with channels, and within channels of (F) hollow sphere at -500 mA.

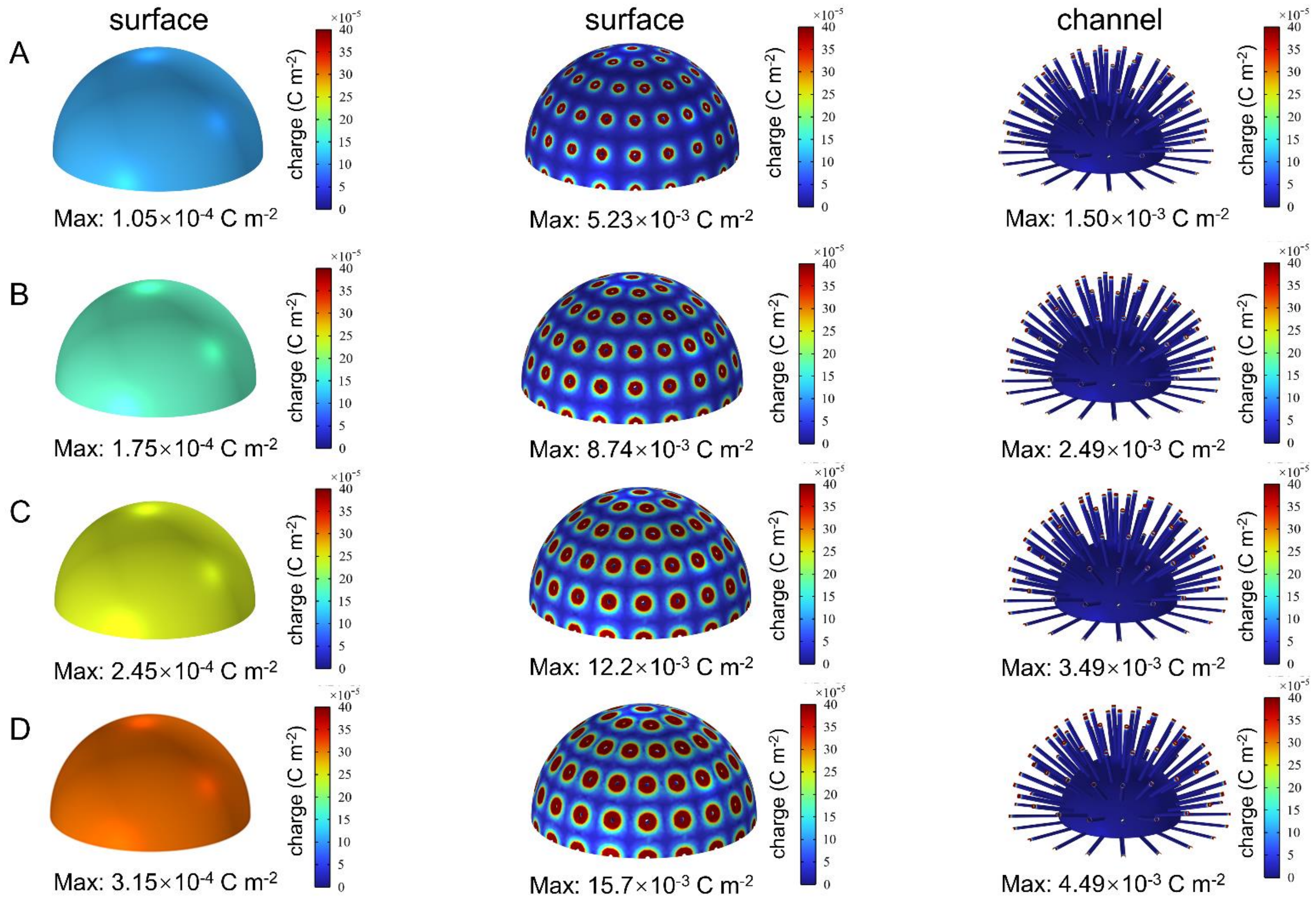

Supplementary Fig. S15 | The negative charge distribution over solid sphere, hollow sphere with channels, and within channels of hollow sphere at (A) -300 mA, (B) -500 mA, (C) -700 mA, and (D) -900 mA.

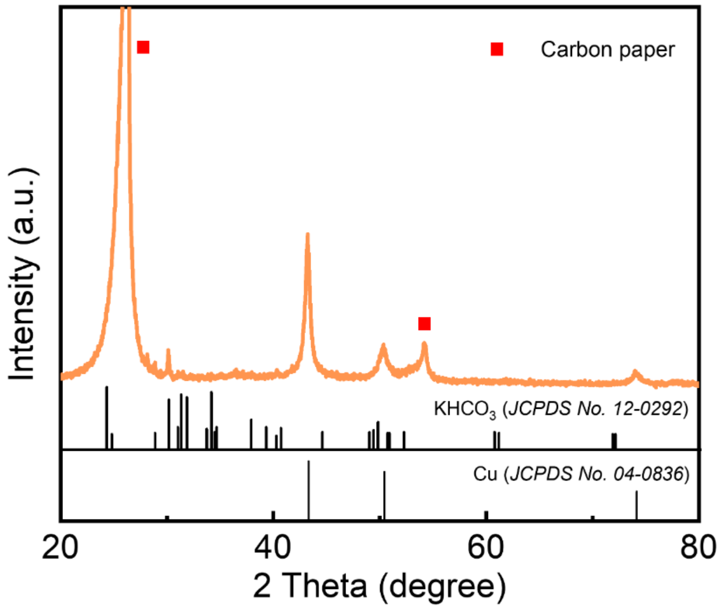

Supplementary Fig. S16 | The XRD patterns of the electrode loaded with La-Cu HS after CO_2 reduction.

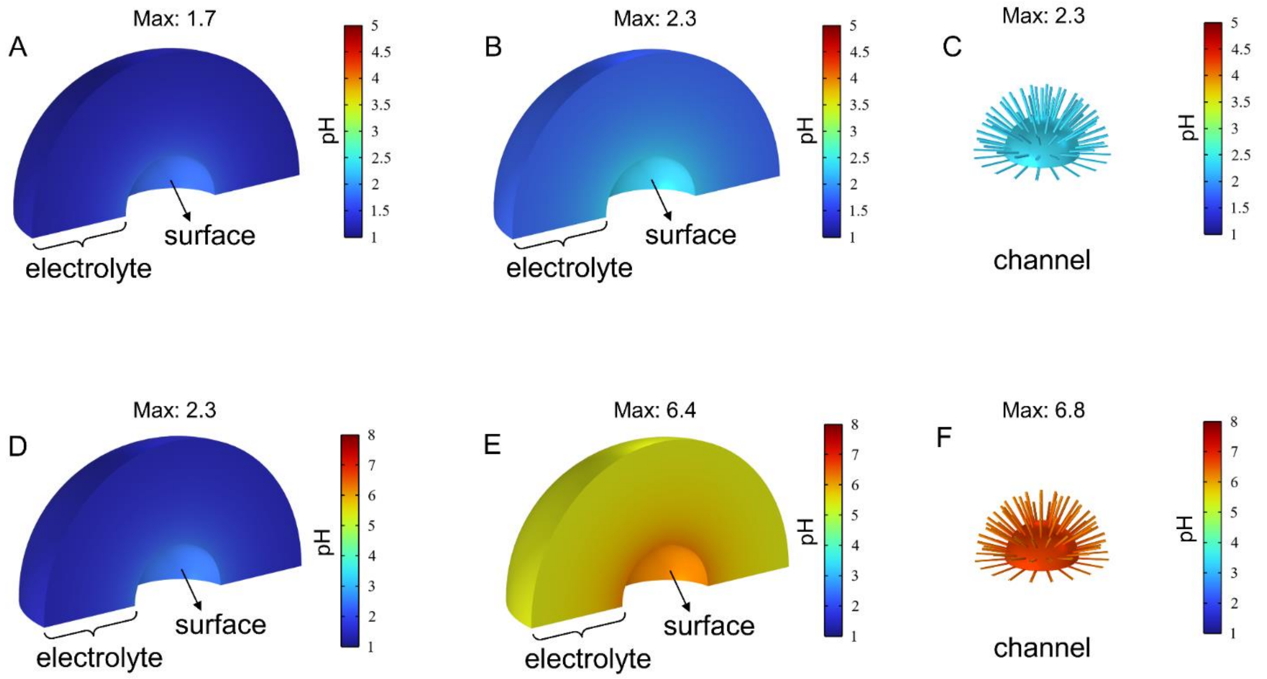

Supplementary Fig. S17 | The pH distribution in electrolyte near surface of (A) solid sphere, (B) hollow sphere with channels, and within (C) channel of hollow sphere at -300 mA. The pH distribution in electrolyte near surface of (D) solid sphere, (E) hollow sphere with channels, and within (F) channels of hollow sphere at -500 mA.

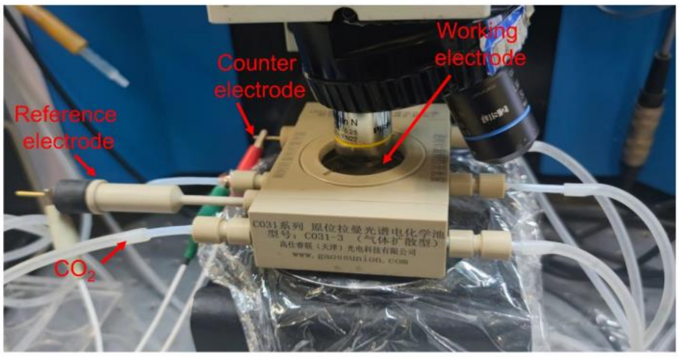

Supplementary Fig. S18 | The photograph of cell used for *in situ* SERS spectroscopy.

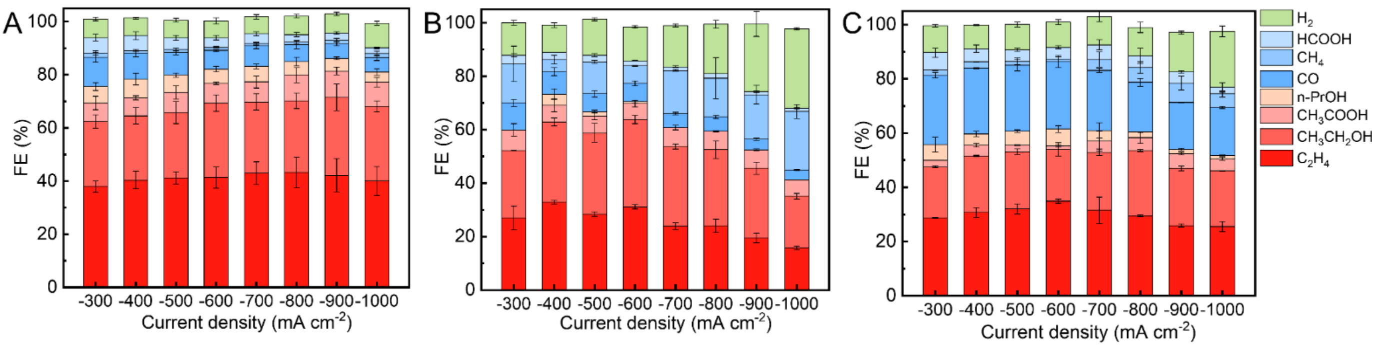

Supplementary Fig. S19 | The product distribution over (A) La-Cu HS, (B) La-Cu SS, and (C) Cu HS under different current densities. Values are means and error bars indicate s.d. (n = 3 replicates).

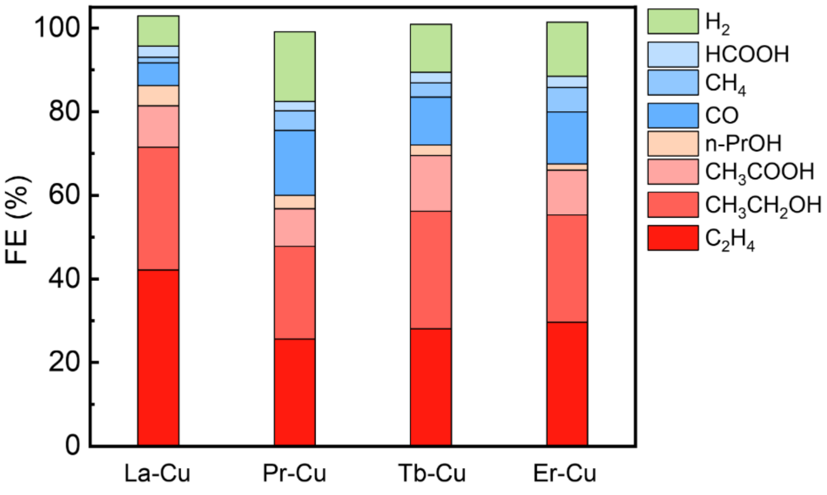

Supplementary Fig. S20 | The products distribution of La-Cu, Pr-Cu, Tb-Cu and Er-Cu at -900 mA cm^{-2}.

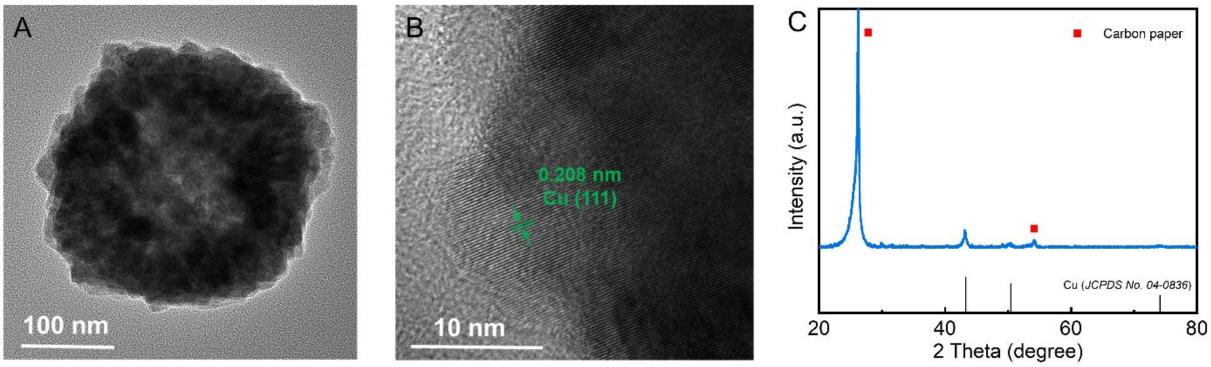

Supplementary Fig. S21 | (A) TEM image, (B) HRTEM image, and (C) XRD pattern of La-Cu HS with La/Cu ratio of 0.1.

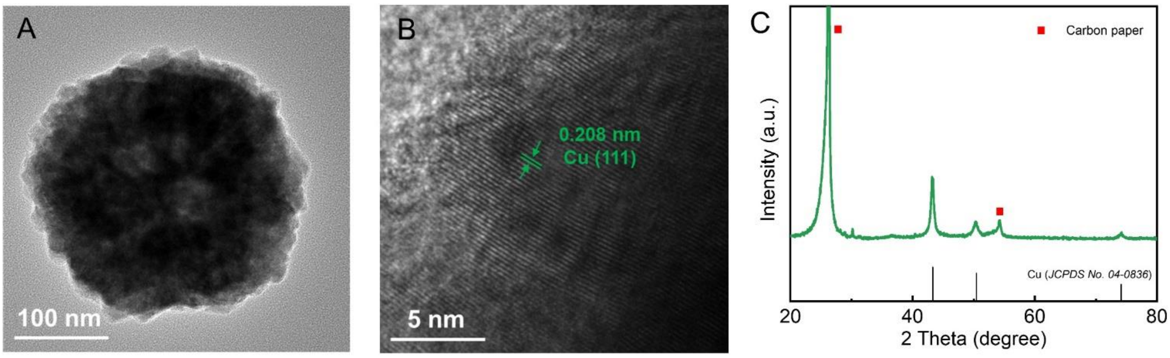

Supplementary Fig. S22 | (A) TEM image, (B) HRTEM image, and (C) XRD pattern of La-Cu HS with La/Cu ratio of 0.2.

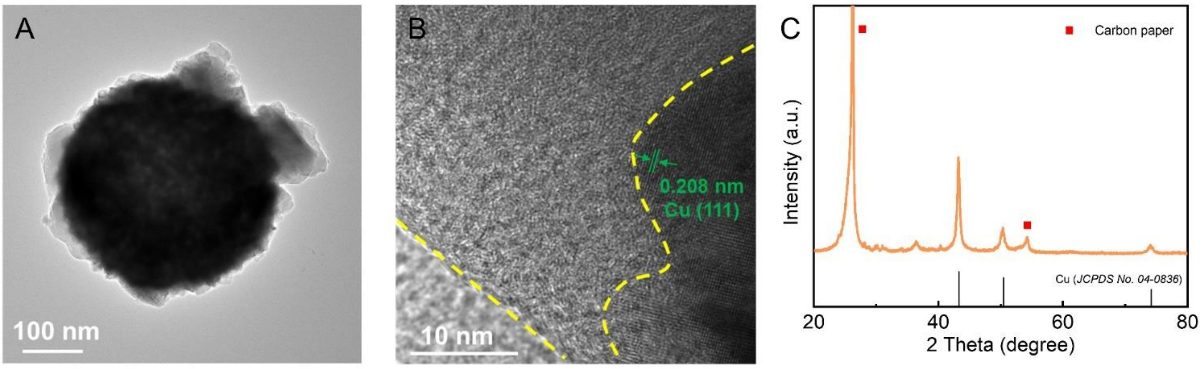

Supplementary Fig. S23 | (A) TEM image, (B) HRTEM image, and (C) XRD pattern of La-Cu HS with La/Cu ratio of 0.4.

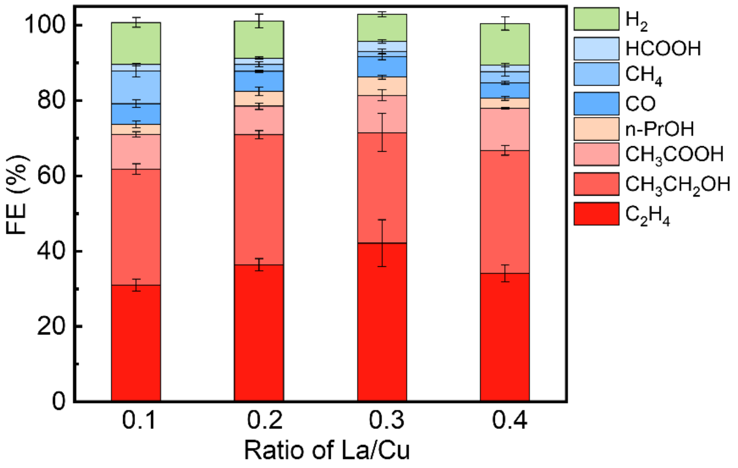

Supplementary Fig. S24 | Product distribution of La-Cu HS with various La/Cu ratio at -900 mA cm^{-2}. Values are means and error bars indicate s.d. (*n* = 3 replicates).

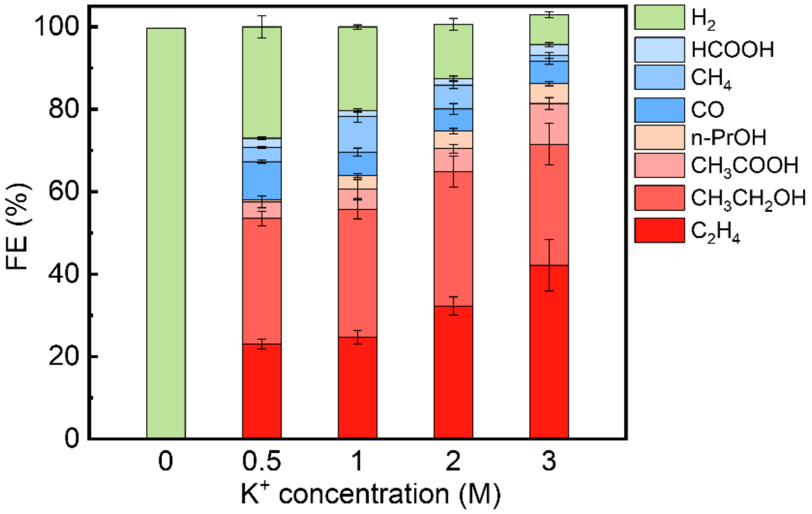

Supplementary Fig. S25 | The products FE of La-Cu HS in 0.05 M H_2SO_4 aqueous solution containing different KCl concentrations at -900 mA cm^{-2}. Values are means and error bars indicate s.d. (*n* = 3 replicates).

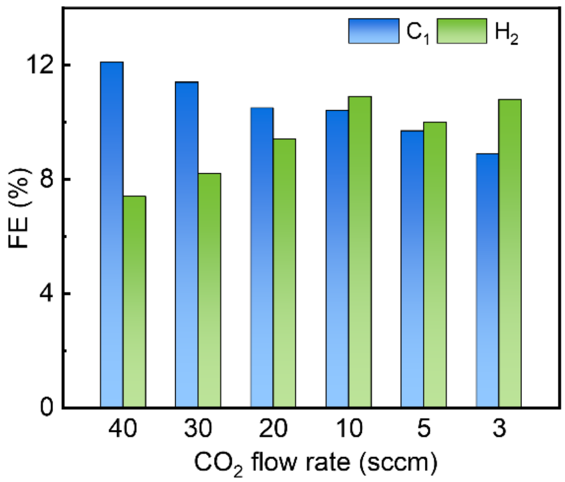

Supplementary Fig. S26 | C_1 products and H_2 FE of La-Cu HS under different CO_2 gas flow rate at -900 mA cm^{-2}

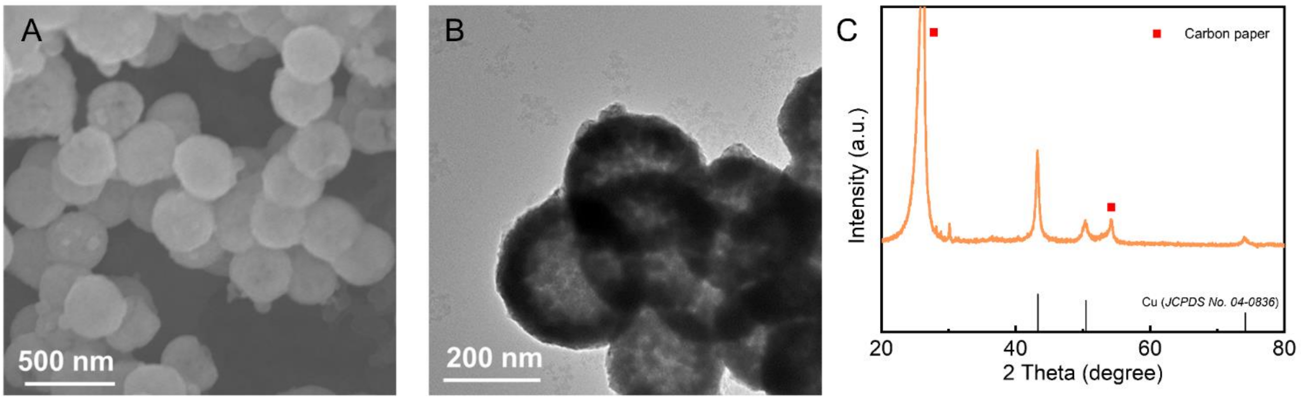

Supplementary Fig. S27 | (A) SEM image, (B) TEM image, and (C) XRD pattern of La-Cu HS after 40 h CO_2RR.

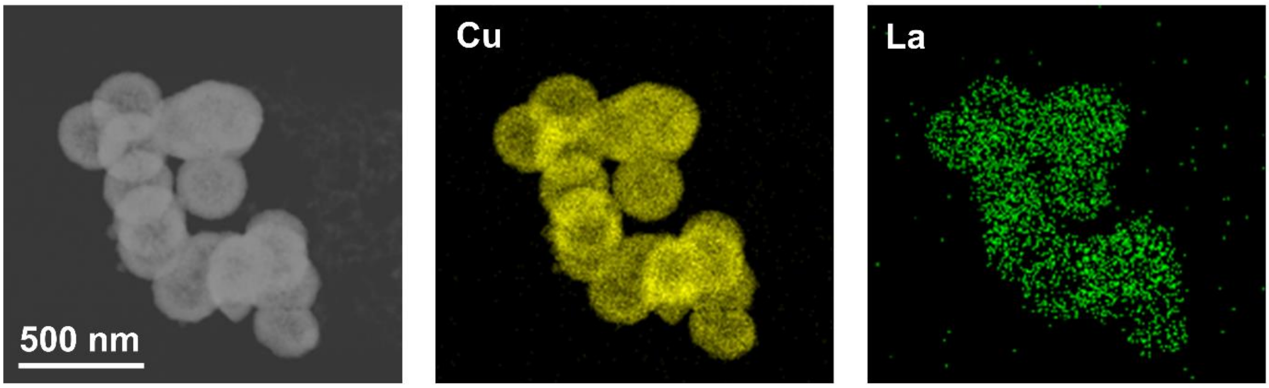

Supplementary Fig. S28 | EDS mapping of La-Cu HS after 40 h CO_2RR. Yellow and green represent Cu and La elements, respectively. The results indicate that Cu and La elements are uniformly distributed on La-Cu HS.

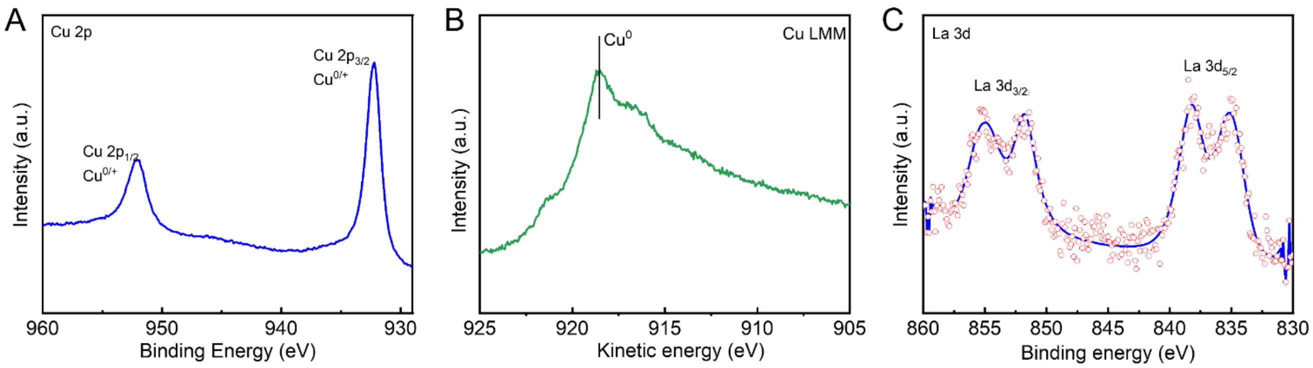

Supplementary Fig. S29 | (A) Cu $2p_{3/2}$ XPS spectrum, (B) Cu Auger LMM spectrum, and (C) La 3d XPS profile of La-Cu HS after 40 h CO_2RR.

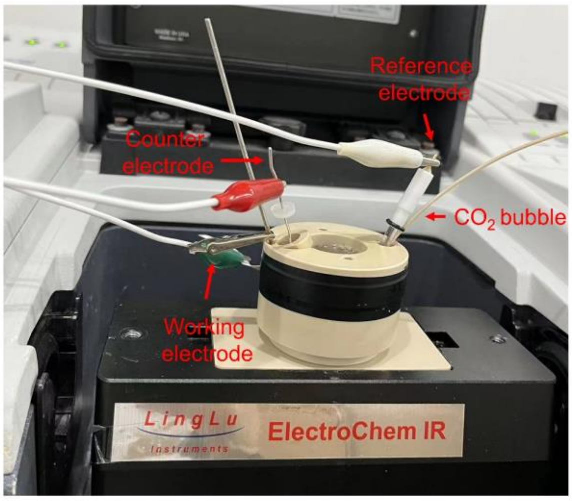

Supplementary Fig. S30 | The photograph and scheme of cell used for *in situ* ATR-SEIRAS spectroscopy.

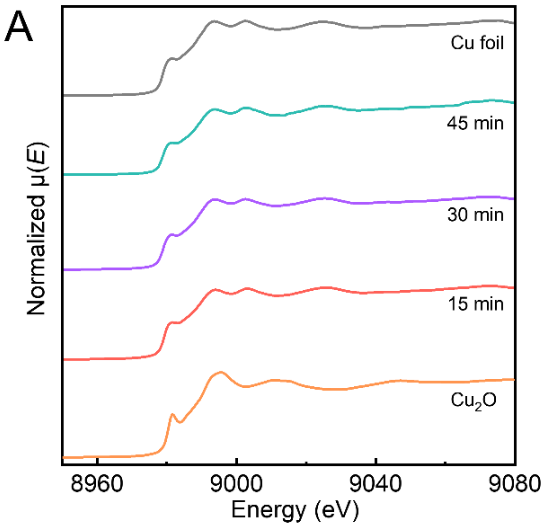

Supplementary Fig. S31 | (A) *In-situ* Cu K-edge XANES spectra and (B) *in-situ* FT EXAFS spectra of La-Cu HS at different reaction time.

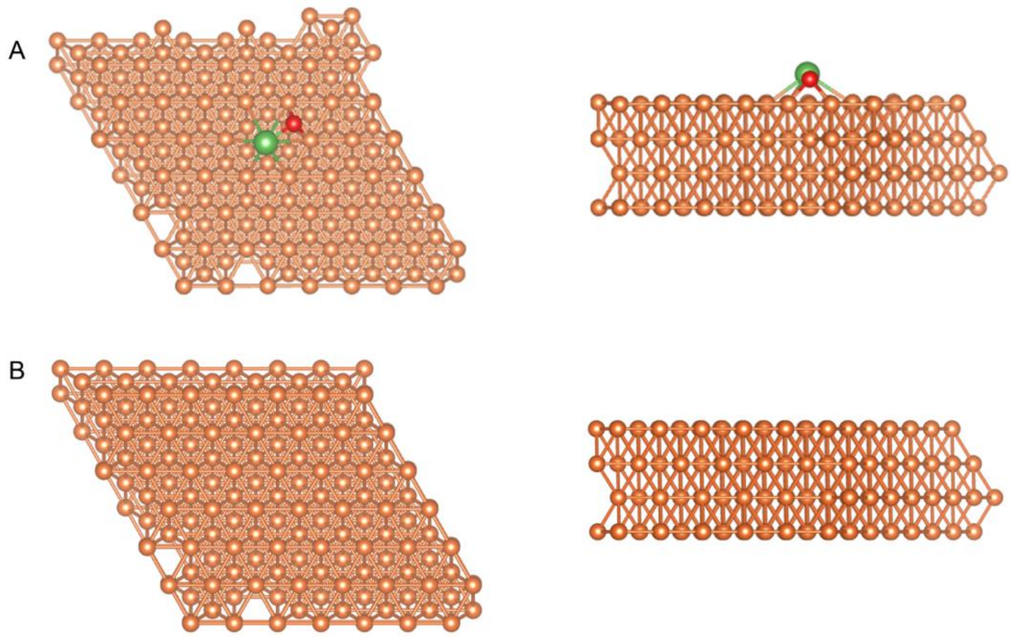

Supplementary Fig. S32 | Top and side view of (A) La-O-Cu (111) and (B) Cu (111) models.

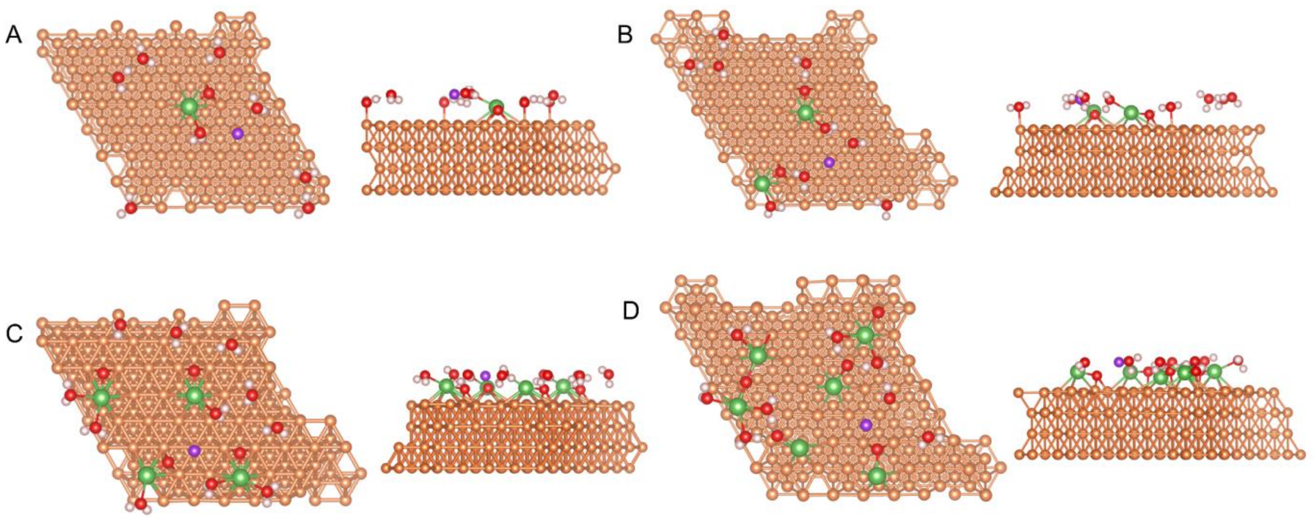

Supplementary Fig. S33 | Top and side view of (A) 1La-Cu, (B) 2La-Cu, (C) 4La-Cu and (4) 6La-Cu models with H_2O and K^+ species on surface. The bronze, green, red, white and purple balls represent Cu, La, O, H and K atoms, respectively.

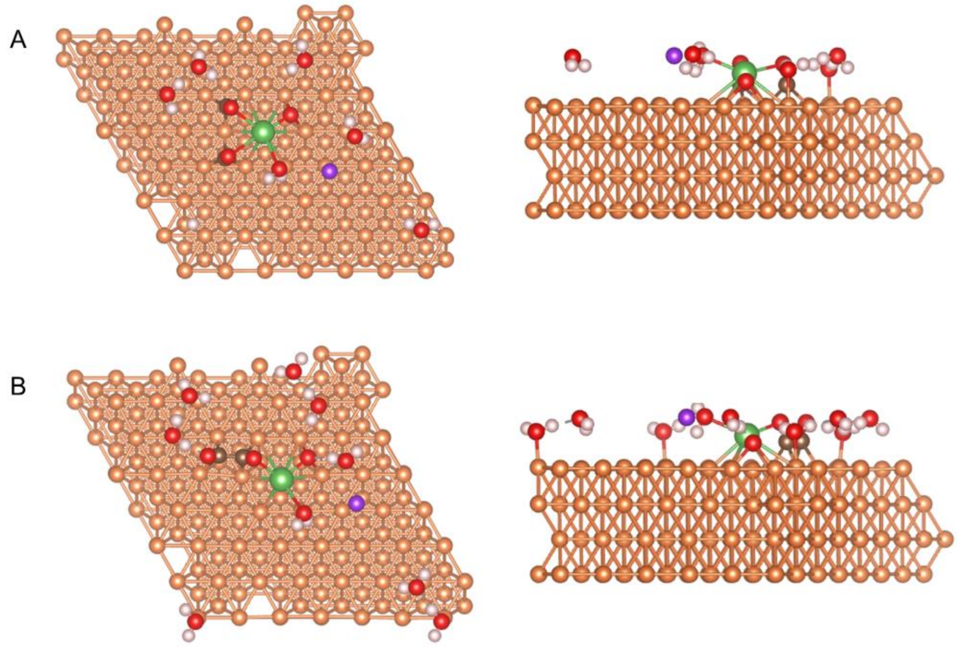

Supplementary Fig. S34 | Top and side view of (A) 2*CO and (B) O*CCO over 1La-Cu model with H_2O and K^+ species on surface. The bronze, green, red, black, white and purple balls represent Cu, La, O, C, H and K atoms, respectively.

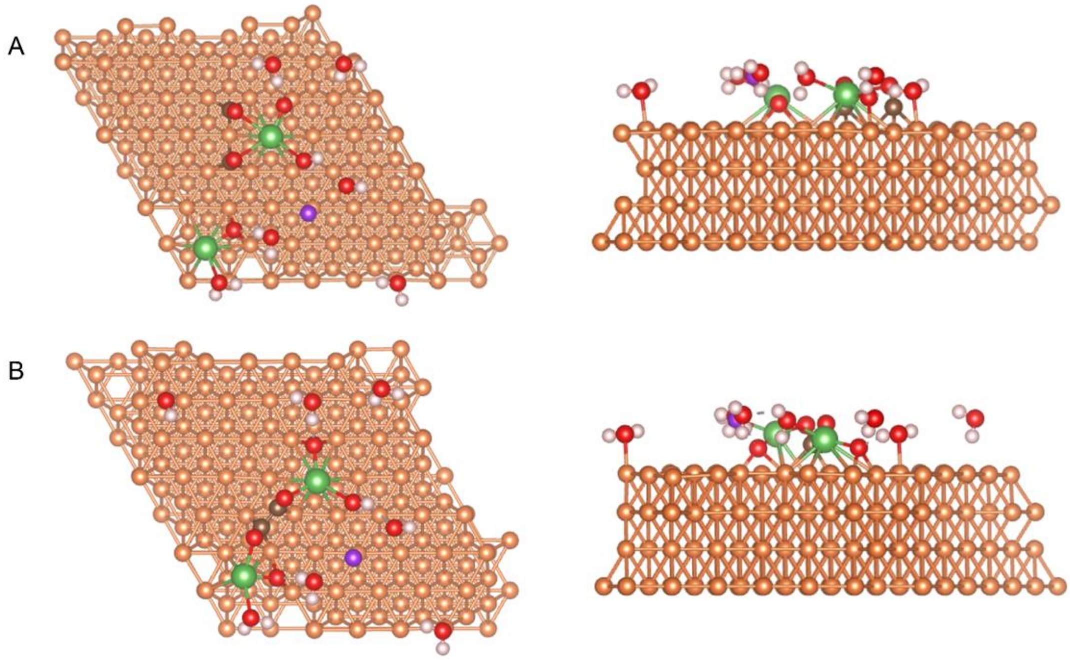

Supplementary Fig. S35 | Top and side view of (A) 2*CO and (B) O*CCO over 2La-Cu model with H_2O and K^+ species on surface. The bronze, green, red, black, white and purple balls represent Cu, La, O, C, H and K atoms, respectively.

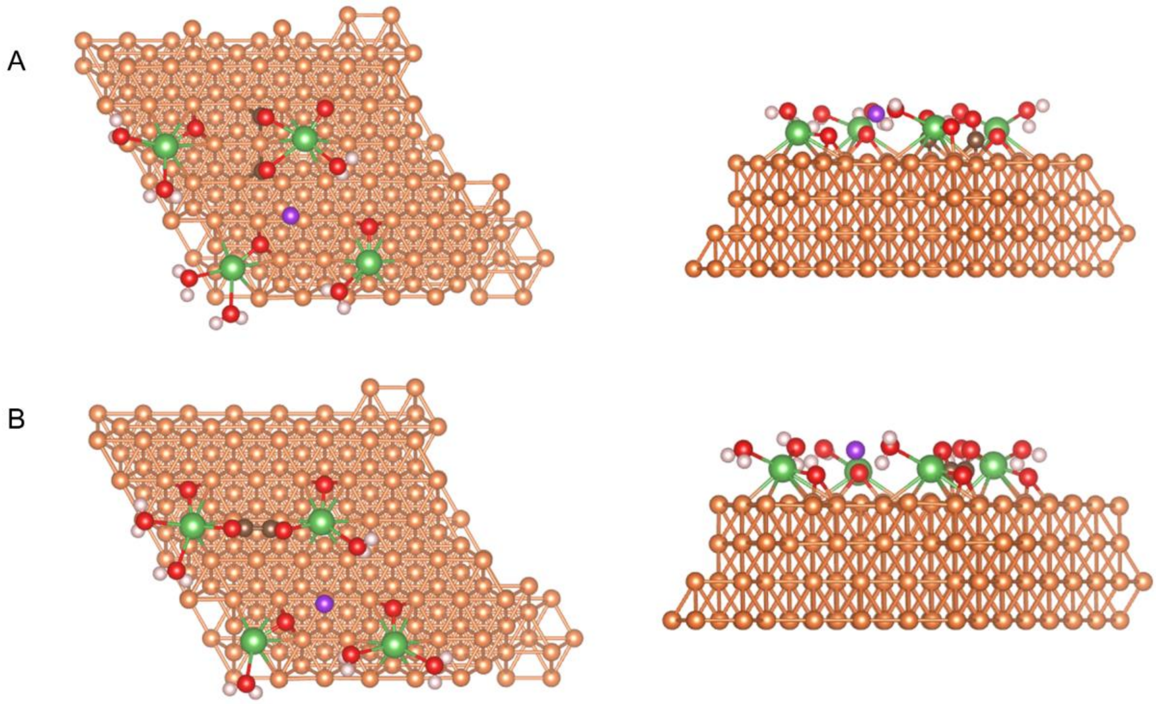

Supplementary Fig. S36 | Top and side view of (A) 2*CO and (B) O*CCO over 4La-Cu model with H_2O and K^+ species on surface. The bronze, green, red, black, white and purple balls represent Cu, La, O, C, H and K atoms, respectively.

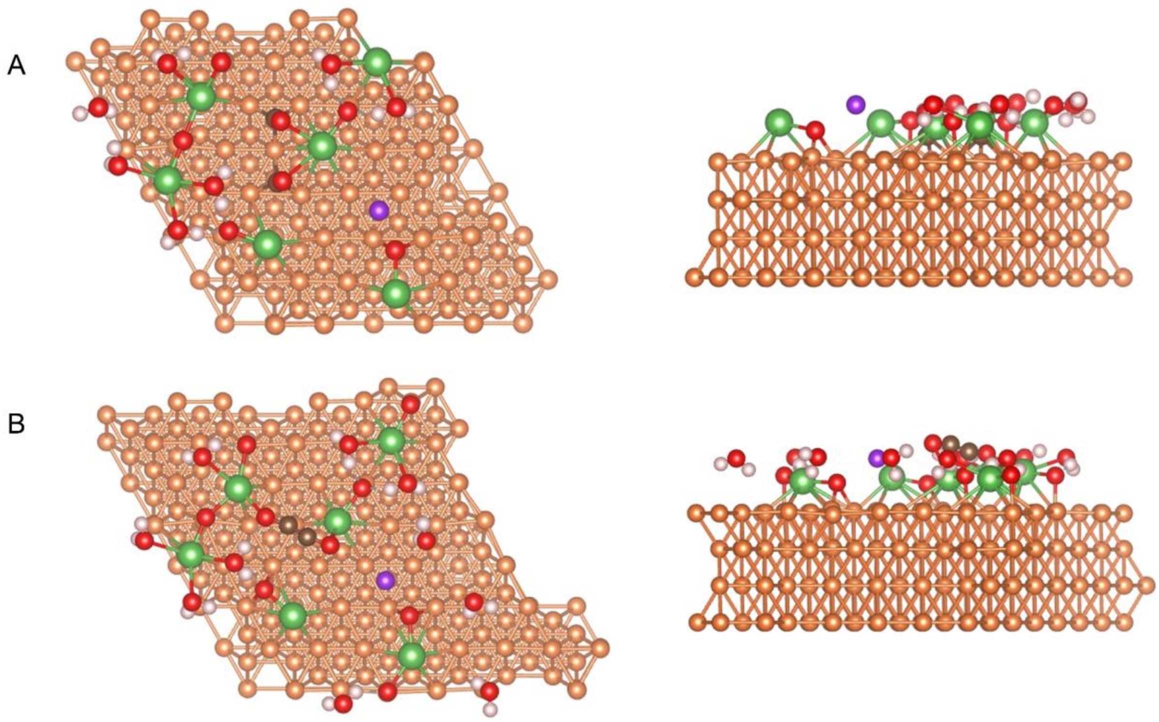

Supplementary Fig. S37 | Top and side view of (A) 2*CO and (B) O*CCO over 6La-Cu model with H_2O and K^+ species on surface. The bronze, green, red, black, white and purple balls represent Cu, La, O, C, H and K atoms, respectively.

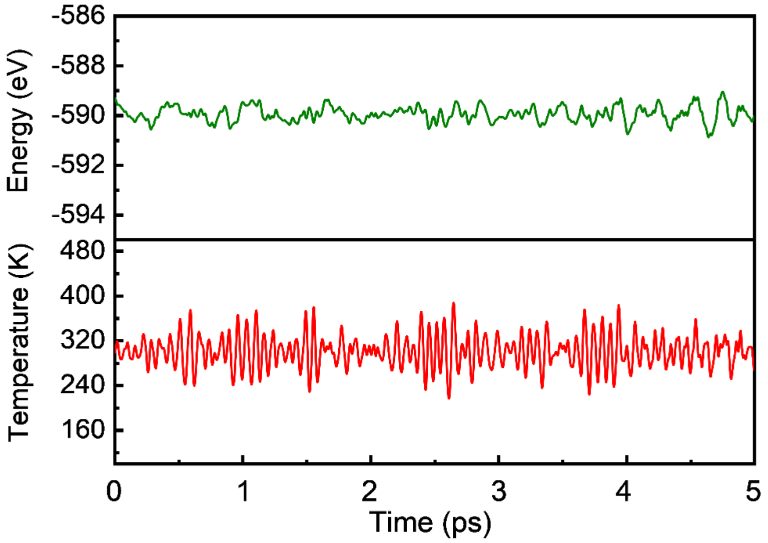

Supplementary Fig. S38 | Energy and temperature variations during the AIMD simulation for 5 ps.

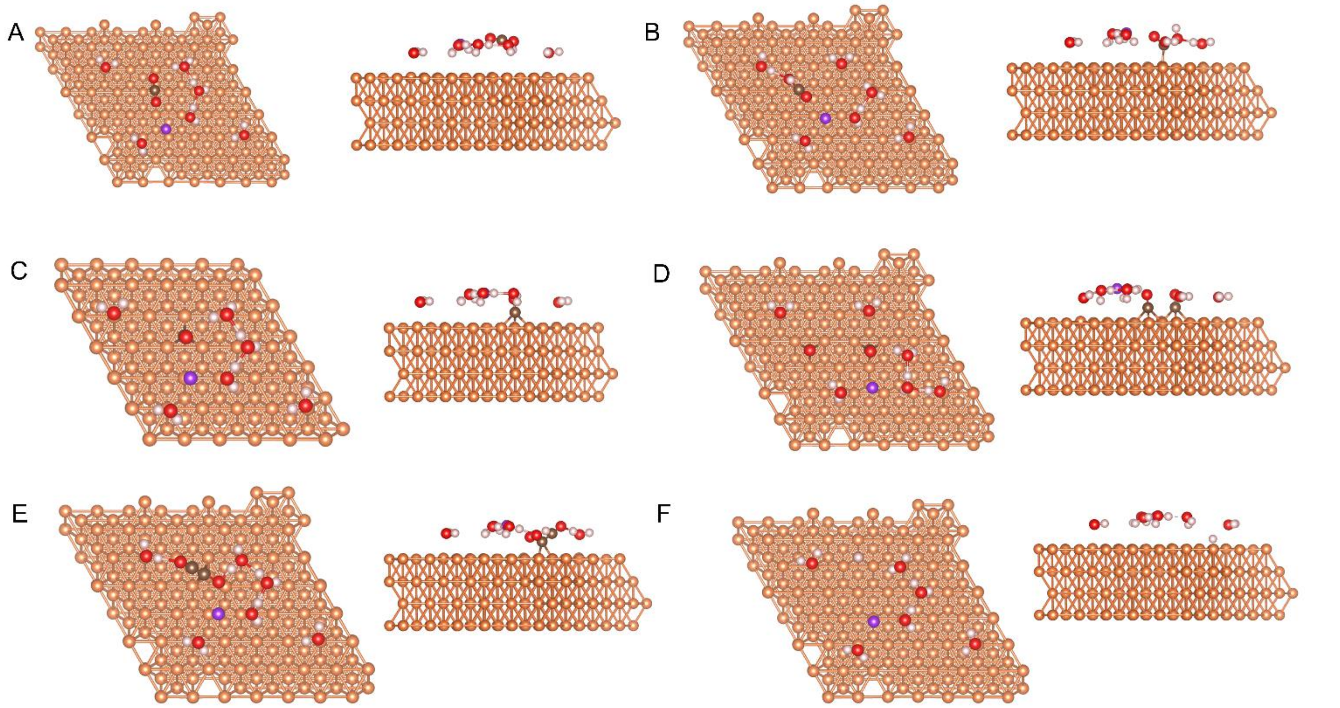

Supplementary Fig. S39 | Top and side view of (A) CO_2, (B) *COOH, (C) *CO, (D) 2*CO, (E) O*CCO and (F) *H over Cu model with H_2O and K^+ species on surface. The bronze, black, red, white and purple balls represent Cu, C, O, H and K atoms, respectively.

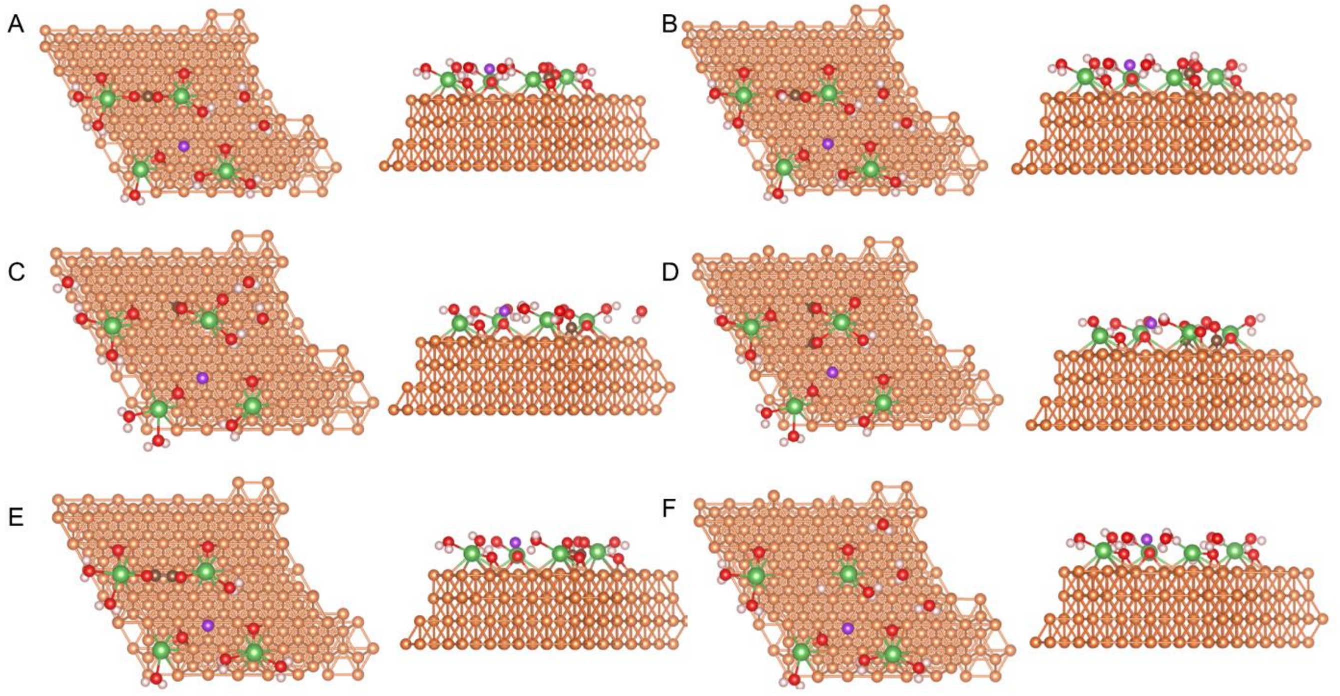

Supplementary Fig. S40 | Top and side view of (A) CO_2, (B) *COOH, (C) *CO, (D) 2*CO, (E) O*CCO and (F) *H over 4La-Cu model with H_2O and K^+ species on surface. The bronze, green, black, red, white and purple balls represent Cu, La, C, O, H and K atoms, respectively.

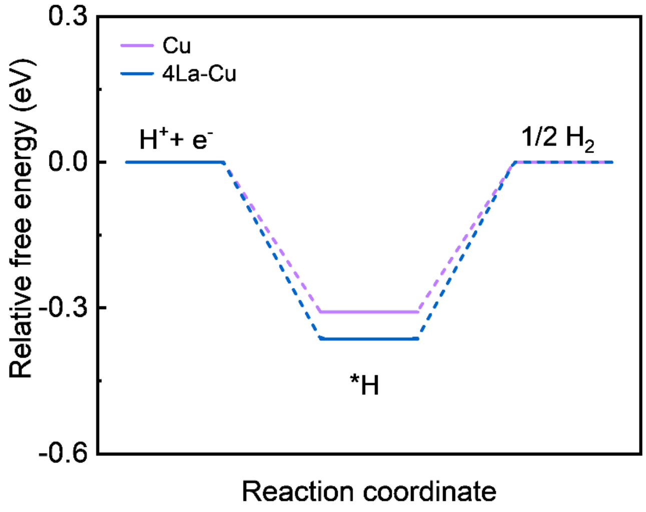

Supplementary Fig. S41 | Calculated free energy diagram for HER over Cu and 4La-Cu.

Supplementary Table S1 | Comparison of CO_2RR-to-C_{2+} products over various reported electrocatalysts in acidic electrolyte.

**Catalyst**	**pH**	**C_{2+} FE (%)**	**j_{C2+} (mA cm^{-2})**	**Stability (h)**	**Ref.**
La-Cu HS	<1	86.2	775.8	40	This work
ER-CuNS	<1	84	557	30	[1]
CAL-modified Cu	<1	48	576	10	[2]
Pd-Cu	2	89	500	4.5	[3]
Cu/C	<1	~37	~200	~4	[4]
COF:PFSA-modified PTFE–Cu	<1	75	150	20	[5]
Cu/PTFE	-	55	~55	9	[6]
EC-Cu	<1	90	~180	10	[7]
Carbon/Cu/PTFE	2	64.5*	~200	-	[8]
$Cu_{0.9}Zn_{0.1}$	4	70	210	~35	[9]
CG-medium	<1	~80	~80	160	[10]
Cu/15 mM [tolyl-pyr]	~1	45	~1.1	6	[11]

* Represents ethylene Faradaic efficiency

References

1. Ma, Z. et al. CO_2 electroreduction to multicarbon products in strongly acidic electrolyte via synergistically modulating the local microenvironment. *Nat. Commun.* **13**, 7596 (2022).
2. Huang, J. E. et al. CO_2 electrolysis to multicarbon productsin strong acid. *Science* **372**, 1074-1078 (2021).
3. Xie, Y. et al. High carbon utilization in CO_2 reduction to multi-carbon products in acidic media. *Nat. Catal.* **5**, 564-570 (2022).
4. Gu, J. et al. Modulating electric field distribution by alkali cations for CO_2 electroreduction in strongly acidic medium. *Nat. Catal.* **5**, 268-276 (2022).
5. Zhao, Y. et al. Conversion of CO_2 to multicarbon products in strong acid by controlling the catalyst microenvironment. *Nature Synthesis* **2**, 403-412 (2023).
6. O'Brien, C. P. et al. Single pass CO_2 conversion exceeding 85% in the electrosynthesis of multicarbon products via local CO_2 Regeneration. *ACS Energy Lett.* **6**, 2952-2959 (2021).
7. Cao, Y. et al. Surface hydroxide promotes CO_2 electrolysis to ethylene in acidic conditions. *Nat. Commun.* **14**, 2387 (2023).
8. Wang, Z. et al. Localized alkaline environment via in situ electrostatic confinement for enhanced CO_2-to-ethylene conversion in neutral medium. *J. Am. Chem. Soc.* **145**, 6339-6348 (2023).
9. Zhang, J. et al. Accelerating electrochemical CO_2 reduction to multi-carbon products via asymmetric intermediate binding at confined nanointerfaces. *Nat. Commun.* **14**, 1298 (2023).
10. Fan, M. et al. Cationic-group-functionalized electrocatalysts enable stable acidic CO2 electrolysis. *Nat. Catal.* **6**, 763-772 (2023).
11. Nie, W., Heim, G. P., Watkins, N. B., Agapie, T. & Peters, J. C. Organic additive-derived films on Cu electrodes promote electrochemical CO_2 reduction to C_{2+} products under strongly acidic conditions. *Angew. Chem. Int. Ed.* **62**, e202216102 (2023).
